# Influence of carbon nanotube suspensions on Casson fluid flow over a permeable shrinking membrane: an analytical approach

**DOI:** 10.1038/s41598-023-30482-6

**Published:** 2023-02-27

**Authors:** Rudraiah Mahesh, Ulavathi Shettar Mahabaleshwar, Filippos Sofos

**Affiliations:** 1 Department of Studies in Mathematics, Davangere University, Shivagangothri, Davangere, 577 007 India; 2 Condensed Matter Physics Laboratory, Department of Physics, University of Thessaly, 35100 Lamia, Greece

**Keywords:** Applied mathematics, Mechanical and structural properties and devices

## Abstract

The present work employs the single-wall carbon nanotube (SWCNT) and multiwall carbon nanotube (MWCNT) models on axisymmetric Casson fluid flow over a permeable shrinking sheet in the presence of an inclined magnetic field and thermal radiation. By exploiting the similarity variable, the leading nonlinear partial differential equations (PDEs) are converted into dimensionless ordinary differential equations (ODEs). The derived equations are solved analytically, and a dual solution is obtained as a result of the shrinking sheet. The dual solutions for the associated model are found to be numerically stable once the stability analysis is conducted, and the upper branch solution is more stable compared to lower branch solutions. The impact of various physical parameters on velocity and temperature distribution is graphically depicted and discussed in detail. The single wall carbon nanotubes have been found to achieve higher temperatures compared to multiwall carbon nanotubes. According to our findings, adding carbon nanotubes volume fractions to convectional fluids can significantly improve thermal conductivity, and this can find applicability in real world applications such as lubricant technology, allowing for efficient heat dissipation in high-temperatures, enhancing the load-carrying capacity and wear resistance of the machinery.

## Introduction

The thermal conductivity of nanofluids has been a popular point of research during the last few decades. The majority of these studies focuses on understanding their properties with the goal of enhancing heat transfer in a variety of commercial and technological applications, including nuclear reactors, power production, material processing, bioengineering, transportation, paper cooling and drying, electronics, medical, and food industry^1^. Means of increasing thermal conductivity include the addition of high thermal conductivity materials, usually carbon-based, metal-based, or other structures like silica or boron nitride^2^.

Towards the incorporation of the material that would exhibit excellent thermal properties and mechanical strength^3^, Sumio Iijima^4^ made a pioneering discovery of cylindrical carbon atom structures with sizes ranging from 1 to 100 nanometers, the Carbon Nanotube (CNT). CNTs achieve increased thermal conductivity, strength, stiffness, and present low density and large surface area-to-volume ratio. They can be classified as single-wall (SWCNTs) or multi-wall (MWCNTs), depending on the number of layers they are consisted of. In comparison to other nanofluids, CNT suspensions achieve better thermal characteristics.

Recently, the effect of CNTs on the magnetohydrodynamics (MHD) flow of a Newtonian^5^ and a non-Newtonian^6^ fluid under radiation and mass transpiration over a stretching/shrinking sheet has been under investigation. Hussain et al.^7^ have shown that SWCNTs are capable of achieving higher temperature values compared to MWCNTs, by incorporating the mixed convection and analysing the effect of the radiation, heat generation/absorption and diffusion species with viscous effect. The impact of CNTs on MHD fluid flow with thermal radiation and heat source/sink over a flat plate stretching/shrinking sheet was also examined by Mahabaleshwar et al.^8^. Other current studies are focused on the Darcy-Forchheimer flow^9,10^, by investigating the effect of different nanoparticles on a Riga surface and vertical Cleveland z-staggered cavity, while, Batool et al. ^11^ have investigated the micropolar effect on a nanofluid flow through lid driven cavity.

Due to its wide applicability, boundary layer flow analysis is particularly beneficial from a physical perspective. It should be noticed that boundary layer flow over surfaces greatly differs from free flow regime over stationary plates, as shown by Sakiadis^12,13^ and Crane^14^. However, the flow medium plays a major role in the whole process. Non-Newtonian fluids have gained increased research interest lately, mainly because of their numerous applications in industrial processes. However, their complicated nature, compared to Newtonian fluids, urges the incorporation of strongly coupled and nonlinear equations to approach. Maxwell, Oldroyd-B, and Jeffrey nanofluids^15^, and Williamson nanofluid^16^ are among those exploited for thermal conductivity and viscosity estimation, while the Rabinowitsch fluid has been exploited in peristaltic biological applications^17^. On the other hand, the Casson fluid has been of practical importance and wide application field, capable of acting like an elastic liquid when there is no flow and just a minor shear stress. Casson^18^ pioneered the use of this approach to replicate industrial inks in his work, unblocking the passage to numerous engineering applications^19^ and theoretical studies^20^. Vaidya et al.^21^ have also investigated the consequences of heat transfer on a non-Newtonian fluid flow via a porous axisymmetric tube with slip effects. Similar works focus on the derivation of an analytical solution for non-Newtonian fluid flow through a porous shrinking surface in the presence of radiation at wall temperature^22^, and the analysis of the thermal performance of Fe_3_O_4_ and Cu nanoparticles with blood as the base fluid on a Casson substantial with MHD and Hall current effects^23^.

Magnetic-driven flows are of paramount importance in engineering, biochemistry, medical applications, the petroleum sector, power generators, magnetic drug targeting, and more^24–27^. Thermal radiation has a substantial influence on boundary layer flow in high-temperature processes. As it affects cooling rates, the role of heat radiation is critical for assuring product quality. MHD boundary layer flows involve the application of a magnetic force to electrically conducting fluids. This field generates currents and a Lorentz force opposing to the fields. Devi and Devi^28^ have numerically examined the impact of Lorentz force on a hybrid nanofluid ejected from a stretched sheet. To explore the turbulent force convection heat transport properties of hybrid nanofluid, Shafiq et al.^29^ has depicted the impact of the Marangoni effect on axisymmetric convective flow by adding the additional parameter of inclined MHD in Casson fluid on infinite disk. Flows over porous shrinking surfaces and the respective solutions have been also obtained^30,31^.

On the other hand, Turkyilmazoglu^32^ has given dual solutions for 2D magneto flow in sheets that are constantly contracting or expanding due to a pair stress fluid. Multiple exact solutions of a water-based graphene nanofluid created by porous shrinking or stretching a sheet with a heat sink or source are given by Aly^33^, while Khan et al.^34^ have obtained the closed-form exact analytical solution for the axisymmetric hybrid nanofluid on a permeable non-linear radially shrinking/stretching sheet. Different model geometries include the investigation of Casson fluid flows past a nonlinear permeable stretching cylinder on a porous medium^35^, a cylinder of non-uniform radius^36^, two permeable spheres^37^, and a rotating disk^38^.

The field of investigation is enriched by axisymmetric flow cases where the subsequent conditions vary^39–41^. Turkyilmazoglu has derived the exact solution for the fluid flow problem^42,43^, while Wahid et al.^44^ have approached the analytical solution of hybrid nanofluid flow with partial slip on permeable stretching surface with the effect of radiation. Stability analysis has been extensively utilised to validate the real solution mathematically, first developed by Merkin^45^, and, on this basis, relevant research has covered both steady and unsteady cases^46–48^.

The current study aims to dive deeper towards understanding of the mechanisms involved in Casson fluid flows. The main focus is centred around the derivation of the exact dual solutions in the presence of SWCNTs and MWCNTs, saturated with H_2_O and over a shrinking surface, under the impact of an inclined MHD, radiation and mass transpiration. The exact multiple solutions for velocity and temperature distribution are discussed in detail and are graphically explained. The key concepts of this analytical investigation can be incorporated in various engineering and industrial applications, concerning medical applications, micro-fluidic devices, nanofluid pumps, and space technology. In the next Sections, we discuss the physical model of the problem and the respective mathematical solutions, present the stability analysis, results and validation tests are discussed next, and, finally, the key concepts are summarized in the “Conclusions”Section.

## Methods

### Physical model

**Figure 1:**
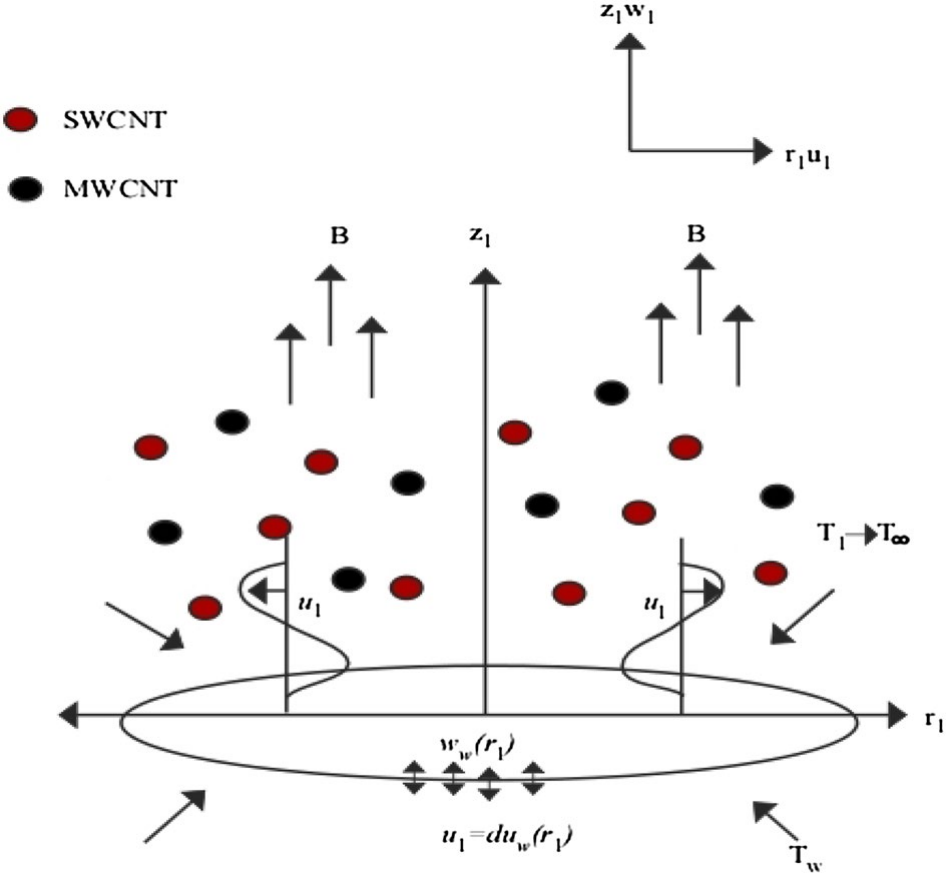
The physical model of the flow.

Physical modelling of the basic equation considers a two-dimensional axisymmetric non-Newtonian fluid containing SWCNTs and MWCNTs with the impact of inclined magnetic field boundary layer flow and thermal radiation on heat ttransformed boundary by a nonlinear permeable radially shrinking surface subjected to the plane $\left({r}_{1},\xi \right)$. Figure 1 presents the physical model on SWCNT and MWCNT nano particles and their cartesian cylindrical coordinates $\left({r}_{1},\xi ,{z}_{1}\right)$, where flow is induced while ${T}_{\infty }<{T}_{w}$. Here, ${r}_{1}$ and ${z}_{1}$ are the coordinates corresponding to the vertical and horizontal direction, and ${T}_{\infty }$ and ${T}_{w}$ the ambient and surface temperature of the sheet, respectively. The sheet surface’s velocity model is assumed to be ${u}_{w}\left({r}_{1}\right)=b{r}_{1}^{3}$, where *b*: constant. The external magnetic field acts in the direction normal to surface as $B\left({r}_{1}\right)={B}_{0}{r}_{1}$. Furthermore, the permeable sheet surface on wall mass transfer of velocity is given by ${w}_{w}\left({r}_{1}\right)=-{r}_{1}{\upsilon }_{0}$, where ${\upsilon }_{0}$ is characterized as sheet porosity. Moreover, the conditions ${w}_{w}<0$ and ${\upsilon }_{0}>0$ are applied to represent the injection, while ${w}_{w}>0$ and ${\upsilon }_{0}<0$ represent mass suction.

The Casson fluid^18^ rheological condition of state is given by the following stress tensor:

1 $$\tau_{ij} = \left\{ {\begin{array}{*{20}c} {\left( {\mu_{b} + \frac{{\tau_{y} }}{{\sqrt {2\pi } }}} \right)2e_{ij} \pi > \pi_{c} ,} \\ {\left( {\mu_{b} + \frac{{\tau_{y} }}{{\sqrt {2\pi } }}} \right)2e_{ij} \pi < \pi_{c} ,} \\ \end{array} } \right.$$

where ${\tau }_{y}$ is the yield stress of non‐Newtonian Casson fluid, ${\mu }_{b}$ the plastic dynamic viscosity, $\pi ={{e}_{i}}_{j}{{e}_{i}}_{j}$, with ${{e}_{i}}_{j}$ the ${\left(i,j\right)}^{\text{th}}$ element of the deformation rate and ${\pi }_{c}$ the critical value of the deformation rate.

The expression for non-Newtonian Casson fluid ${\tau }_{y}$ yield stress is

2 $$\tau_{y} = \frac{{\mu_{b} \sqrt {2\pi } }}{\Lambda }$$

When $\pi > \pi_{c}$, a Casson fluid flow occurs, and it is possible to state that

3 $$\mu = \left( {\mu_{b} + \frac{{\tau_{y} }}{{\sqrt {2\pi } }}} \right)$$

By substituting Eq. (2) into Eq. (3), we obtain

4 $$\mu = \mu_{b} \left( {1 + \frac{1}{\Lambda }} \right)$$

On the basis of the above information, the convection term in the momentum equation is written as

5 $$\frac{1}{\rho }\frac{\partial }{\partial y}\left( {\tau_{y} } \right) = \frac{{\mu_{b} }}{\rho }\left( {1 + \frac{1}{\Lambda }} \right)\frac{{\partial^{2} u}}{{\partial y^{2} }} = \upsilon \left( {1 + \frac{1}{\Lambda }} \right)\frac{{\partial^{2} u}}{{\partial y^{2} }}$$

For CNTs we write the equation as ${\upsilon }_{nf}\left(1+\frac{1}{\Lambda }\right)\frac{{\partial }^{2}u}{\partial {y}^{2}}$, where ${\upsilon }_{nf}=\frac{{\mu }_{nf}}{{\rho }_{nf}}$,

The leading governing equations are (see Ref.^34^ for details)

6 $$\frac{{\partial u_{1} }}{{\partial r_{1} }} + \frac{{u_{1} }}{{r_{1} }} + \frac{{\partial w_{1} }}{{\partial z_{1} }} = 0.$$

7 $$u_{1} \frac{{\partial u_{1} }}{{\partial r_{1} }} + w_{1} \frac{{\partial u_{1} }}{{\partial z_{1} }} = \frac{{\mu_{nf} }}{{\rho_{nf} }}\left( {1 + \frac{1}{\Lambda }} \right)\frac{{\partial^{2} u_{1} }}{{\partial z_{1}^{2} }} - \frac{{\sigma_{nf} B^{2} \left( r \right)}}{{\rho_{nf} }}\sin \left( \tau \right)u_{1} .$$

8 $$u_{1} \frac{{\partial T_{1} }}{{\partial r_{1} }} + w_{1} \frac{{\partial T_{1} }}{{\partial z_{1} }} = \frac{{k_{nf} }}{{\left( {\rho Cp} \right)_{nf} }}\frac{{\partial^{2} T_{1} }}{{\partial z_{1}^{2} }} - \frac{1}{{\left( {\rho Cp} \right)_{nf} }}\frac{{\partial q_{r} }}{{\partial z_{1} }}.$$

where ${u}_{1}$ and ${w}_{1}$ are the velocity components with respect to direction ${r}_{1}$ and ${z}_{1}$, ${T}_{1}$ the fluid temperature, ${\mu }_{nf}$,${\rho }_{nf}$, ${\sigma }_{nf}$, ${\left(\rho Cp\right)}_{nf}$ are the dynamic viscosity, density, electrical conductivity, and specific heat of the nano fluid, respectively. The first terms on the right-hand side of Eq. (7) represent the non-Newtonian Casson fluid, and the second term of Eqs. (7) and (8) represents the inclined magnetic field and thermal radiation which can take as radioactive heat flux ${q}_{r}$, respectively.

These equations are subject to the boundary condition:

9 $$\left\{ \begin{gathered} u_{1} = du_{w} \left( {r_{1} } \right),\quad w_{1} = w_{w} \left( {r_{1} } \right),\quad T_{1} = T_{w} \,\,{\text{at}}\quad {\text{z}}_{{1}} = 0, \hfill \\ u_{1} \to 0,\,\,\,\,\quad T_{1} \to T_{\infty } ,\,\,{\text{as}}\quad {\text{z}}_{{1}} \to \infty . \hfill \\ \end{gathered} \right.$$

where ${u}_{1}$ and ${w}_{1}$ are the velocity components with respect to direction ${r}_{1}$ and ${z}_{1}$, ${T}_{1}$ the fluid temperature, ${u}_{w}\left({r}_{1}\right)$ and ${w}_{w}\left({r}_{1}\right)$ represents the surface’s velocity and wall mass transfer of velocity of the sheet, ${T}_{\infty }$ and ${T}_{w}$ the ambient and surface temperature of the sheet, respectively, $d$ represents the stretching $\left(d>0\right)$, shrinking $\left(d<0\right)$ and static $\left(d=0\right)$ sheet. For the present work we consider only shrinking $\left(d<0\right)$ case.

The radioactive heat flux ${q}_{r}$ is added to the model by using Rosseland approximations and can be written in the following simplified form, as

10 $$q_{r} = \frac{{ - 16\sigma^{*} T_{\infty }^{3} }}{{3k^{*} }}\frac{{\partial T_{1}^{4} }}{{\partial z_{1} }}$$

Then, the term $T_{1}^{4}$ is expanded by using Taylor’s series and ignoring higher order terms to obtain

11 $$T_{1}^{4} = T_{1\infty }^{4} + 4T_{1\infty }^{4} \left( {T_{1} - T_{1\infty } } \right) + 6T_{1\infty }^{2} \left( {T_{1} - T_{1\infty } } \right)^{2} + ............$$

$T_{1}^{4}$ can be assessed by:

12 $$T_{1}^{4} \cong - 3T_{{1_{\infty } }}^{4} + 4T_{{1_{\infty } }}^{3} T_{1} .$$

where ${\sigma }^{*}$ is the Stefan-Boltzmann constant and ${k}^{*}$ the mean absorption coefficient of base fluid. From Eqs. (8) and (9) we obtain

13 $$u_{1} \frac{{\partial T_{1} }}{{\partial r_{1} }} + w_{1} \frac{{\partial T_{1} }}{{\partial z_{1} }} = \frac{{k_{f} }}{{\left( {\rho Cp} \right)_{nf} }}\left( {\frac{{k_{nf} }}{{k_{f} }} + \frac{{16\sigma^{*} T_{\infty }^{3} }}{{3k^{*} k_{f} }}} \right)\frac{{\partial^{2} T_{1} }}{{\partial z_{1}^{2} }}.$$

### Thermophysical expressions and properties of the CNTs

**Figure 2:**
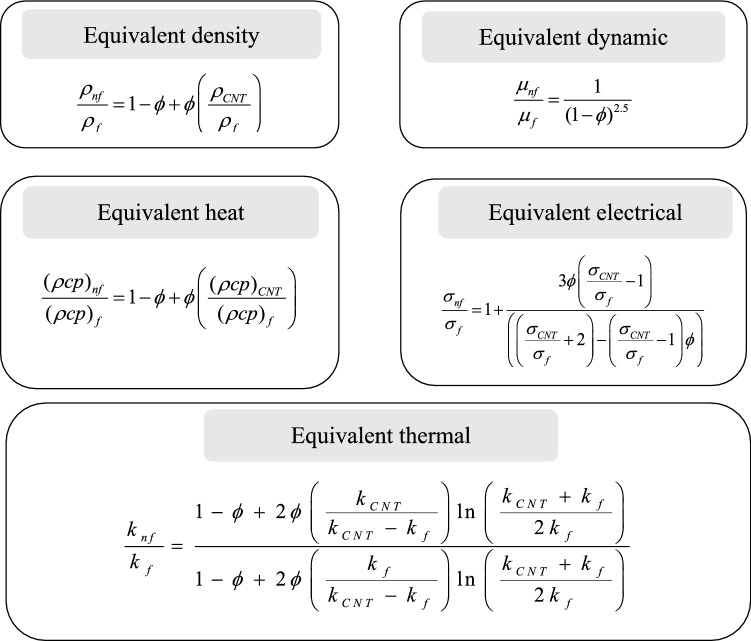
Thermophysical expressions for CNTs in our model.

In Fig. 2, $\phi$ is known as sloid volume fraction of nanoparticle, and with the *nf* as subscript, ${\rho }_{nf},{\mu }_{nf},{\sigma }_{nf},{\kappa }_{nf},\rho C{p}_{nf}$ are the density, viscosity, electrical conductivity, thermal conductivity, heat capacity of the nanofluid, respectively. The *f* subscript denotes the same quantities, but for the base fluid, while the CNT as subscript refers to the carbon nanofluid. The experimental values of $Cp$ (specific heat), $\rho$(density), $\kappa$ (thermal conductivity), and $\sigma$ (electrical conductivity) for the base fluid (water), SWCNT and MWCNT are presented in Table 1^6,7^

**Table 1:** Thermal properties of base fluid and nanoparticles.

Physical properties	Fluid phase (water)	SWCNT	MWCNT
${C}_{p}\left({\mathrm{Jkg}}^{-1}{\mathrm{K}}^{-1}\right)$	4179	425	796
$\rho \left({\mathrm{kgm}}^{-3}\right)$	997.1	2600	1600
$\kappa \left({\mathrm{Wm}}^{-1}{\mathrm{K}}^{-1}\right)$	0.613	6600	3000
$\sigma {\left(\Omega /\mathrm{m}\right)}^{-1}$	0.05	$1\times 1{0}^{7}$	$1\times 1{0}^{7}$

### Similarity variable

We proposed the following similarity variable to simplify the problem,

14 $$\xi = \sqrt {\frac{b}{{\upsilon_{f} }}} z_{1} r_{1} ,\theta \left( \xi \right) = \frac{{T_{1} - T_{\infty } }}{{T_{w} - T_{\infty } }},\psi = - \sqrt {b\upsilon_{f} } r_{1}^{3} f\left( \xi \right)$$

where $\psi$ is the stream function, while $u_{1} \,{\text{and}}\,w_{1}$ are given by $u_{1} = \frac{1}{{r_{1} }}\frac{\partial \psi }{{\partial z_{1} }}$ and $w_{1} = - \frac{1}{{r_{1} }}\frac{\partial \psi }{{\partial r_{1} }}$. Taking Eq. (9) into consideration, the velocity components can be written as

15 $$u_{1} = br_{1}^{3} F^{\prime}\left( \xi \right)\;{\text{and}}\;w_{1} = - \sqrt {b\upsilon_{f} } r_{1} \left( {3F\left( \xi \right) + \xi F^{\prime}\left( \xi \right)} \right)$$

Next, through similarity transformation and thermophysical expressions, Eqs. (7) and (13) are converted into

16 $$A_{1} \left( {1 + \frac{1}{\Lambda }} \right)F^{\prime \prime \prime } - A_{5} M\sin \left( \tau \right)F^{\prime } + A_{2} \left( {3FF^{\prime } - 3F^{\prime 2} } \right) = 0.$$

17 $$\left( {A_{4} + N_{r} } \right)\theta ^{\prime\prime} + 3A_{3} \Pr F\theta ^{\prime} = 0.$$

subjected to boundary conditions as follows:

18 $$\left\{ \begin{gathered} F^{\prime}\left( \xi \right) = d,\quad F\left( \xi \right) = V_{c} ,\quad \theta \left( \xi \right) = 1,\quad {\text{at}}\quad \xi = 0, \hfill \\ F^{\prime}\left( \xi \right) \to 0,\quad \theta \left( \xi \right) = 0,\quad {\text{as}}\quad \xi \to \infty . \hfill \\ \end{gathered} \right.$$

where,

19 $$\left\{ \begin{gathered} A_{1} = \frac{{\mu_{nf} }}{{\mu_{f} }},\quad A_{2} = \frac{{\rho_{nf} }}{{\rho_{f} }},\quad A_{3} = \frac{{\left( {\rho C_{p} } \right)_{nf} }}{{\left( {\rho C_{p} } \right)_{f} }},\quad A_{4} = \frac{{k_{nf} }}{{k_{f} }},\quad A_{5} = \frac{{\sigma_{nf} }}{{\sigma_{f} }}, \hfill \\ M = \frac{{\sigma_{f} B_{0}^{2} }}{{\rho_{f} b}},\quad Nr = \frac{{16\sigma^{*} T_{\infty }^{3} }}{{3k_{f} k^{*} }},\quad Pr = \frac{{(\rho C_{p} )_{f} \nu_{f} }}{{k_{f} }}. \hfill \\ \end{gathered} \right.$$

### Engineering physical quantities of interest

The shear stress coefficient ${C}_{f}$ and the local heat transfer number $N{u}_{{z}_{1}}$ are

20 $$C_{f} = \frac{{2\mu_{nf} \left( {1 + \frac{1}{\Lambda }} \right)}}{{\rho_{f} u_{w}^{2} }}\left( {\frac{{\partial u_{1} }}{{\partial z_{1} }}} \right)_{{z_{1} = 0}} {, }Nu_{{z_{1} }} = \frac{{r_{1} k_{nf} }}{{k_{f} \left( {T_{w} - T_{\infty } } \right)}}\left( {\frac{{\partial T_{1} }}{{\partial z_{1} }}} \right)_{{z_{1} = 0}}$$

By imposing the similarity transformation, we obtain

21 $$0.5{\text{Re}}_{{r_{1} }}^{1/2} C_{f} = \frac{{\mu_{nf} \left( {1 + \frac{1}{\Lambda }} \right)}}{{\mu_{f} }}F^{\prime\prime}\left( 0 \right), {\text{Re}}_{{r_{1} }}^{ - 1/2} Nu_{{z_{1} }} = - \frac{{k_{nf} }}{{k_{f} }}\theta ^{\prime}\left( 0 \right)$$

where ${\mathit{Re}}_{{r}_{1}}^{1/2}=\frac{{u}_{w}\left({r}_{1}\right){r}_{1}}{{\upsilon }_{f}}$ is the Reynolds number.

### Exact solution for momentum equation

The exact analytical solution of Eq. (16) subject to boundary condition from Eq. (18) is

22 $$F\left( \xi \right) = V_{c} + \frac{d}{\beta }\left( {1 - Exp\left[ { - \beta \xi } \right]} \right).$$

By substituting into Eq. (16) and, also, by considering $\tau = \frac{\pi }{2},$ it is $\sin \left( \tau \right) = 1$ . We obtain an algebraic quadratic Equation as

23 $$A_{1} \left( {1 + \frac{1}{\Lambda }} \right)\beta^{2} - 3A_{2} V_{c} \beta - \left( {A_{5} M + 3A_{2} d} \right) = 0.$$

where the roots are

24 $$\beta = \frac{{3V_{c} A_{2} \pm \sqrt {9V_{c}^{2} A_{2}^{2} - 4\left( {1 + \frac{1}{\Lambda }} \right)A_{1} \left( { - 3dA_{2} - MA_{5} } \right)} }}{{2\left( {1 + \frac{1}{\Lambda }} \right)A_{1} }}$$

From Eq. (21), it is clearly shown that the problem has both a lower and an upper branch solution. The velocity component $F^{\prime}\left( \xi \right)$ and the skin friction component $F^{\prime\prime}\left( 0 \right)$ are given by

25 $$F^{\prime}\left( \xi \right) = dExp\left[ { - \beta \xi } \right]\;{\text{and}}\;F^{\prime\prime}\left( 0 \right) = - d\beta$$

### Exact solution for energy equation

Equation (17) can be modified with aid of Eq. (22), and, by introducing a new parameter $\eta =\frac{Pr}{{\beta }^{2}\left[-\beta \xi \right]}$, it transforms to

26 $$\left( {A_{4} + N_{r} } \right)\eta \frac{{d^{2} \theta }}{{d\eta^{2} }} + \left\{ {\left( {A_{4} + N_{r} } \right) + A_{4} \left( {3d\eta - 3\frac{Pr}{{\beta^{2} }}\left( {d + V_{c} \beta } \right)} \right)\frac{d\theta }{{d\eta }}} \right\}$$

with boundary conditions

27 $$\theta \left( {\frac{\Pr }{{\beta^{2} }}} \right) = 1,\quad {\text{and}}\quad \theta \left( 0 \right) = 0.$$

Thus, the outcome of Eq. (26) subject to boundary condition from Eq. (27) in terms of incomplete Gamma function is

28 $$\theta \left( \eta \right) = \frac{{\Gamma \left( {j,o} \right) - \Gamma \left( {j,\frac{{3A_{3} d}}{{\left( {A_{4} + N_{r} } \right)}}\eta } \right)}}{{\Gamma \left( {j,o} \right) - \Gamma \left( {j,\frac{{3A_{3} dPr}}{{\left( {A_{4} + N_{r} } \right)\beta^{2} }}} \right)}} .$$

where $$j=\frac{3{A}_{3}\left(\mathit{Pr}d+{V}_{c}\mathit{Pr}\beta \right)}{\left({A}_{4}+{N}_{r}\right){\beta }^{2}}.$$

In terms of the similarity variable $\xi$, Eq. (28) transforms to

29 $$\theta \left( \xi \right) = \frac{{\Gamma \left( {j,o} \right) - \Gamma \left( {j,\frac{{3A_{3} d}}{{\left( {A_{4} + N_{r} } \right)}}\frac{\Pr }{{\beta^{2} }}Exp\left[ { - \beta \xi } \right]} \right)}}{{\Gamma \left( {j,o} \right) - \Gamma \left( {j,\frac{{3A_{3} dPr}}{{\left( {A_{4} + N_{r} } \right)\beta^{2} }}} \right)}}.$$

Then,

30 $$\theta ^{\prime}\left( \xi \right) = \frac{{ - 3^{j} Exp\left[ { - \left( {\frac{{3A_{3} d\Pr }}{{\left( {A_{4} + N_{r} } \right)\beta^{2} }}Exp\left[ { - \beta \xi } \right]} \right)} \right]\beta \left( {\frac{{3A_{3} d\Pr }}{{\left( {A_{4} + N_{r} } \right)\beta^{2} }}Exp\left[ { - \beta \xi } \right]} \right)^{j} }}{{\Gamma \left( {j,o} \right) - \Gamma \left( {j,\frac{{3A_{3} dPr}}{{\left( {A_{4} + N_{r} } \right)\beta^{2} }}} \right)}}.$$

where $$j=\frac{3{A}_{3}\left(\mathit{Pr}d+{V}_{c}\mathit{Pr}\beta \right)}{\left({A}_{4}+{N}_{r}\right){\beta }^{2}}.$$ and

31 $$- \theta ^{\prime}\left( 0 \right) = \frac{{3^{j} Exp\left[ { - \left( {\frac{{3A_{3} d\Pr }}{{\left( {A_{4} + N_{r} } \right)\beta^{2} }}} \right)} \right]\beta \left( {\frac{{3A_{3} d\Pr }}{{\left( {A_{4} + N_{r} } \right)\beta^{2} }}} \right)^{j} }}{{\Gamma \left( {j,o} \right) - \Gamma \left( {j,\frac{{3A_{3} dPr}}{{\left( {A_{4} + N_{r} } \right)\beta^{2} }}} \right)}}.$$

Equation (31) is the local heat transfer rate at the wall sheet surface.

### Stability analysis

A complete study of the stability of dual solutions for the Eqs. (16-17) along with the boundary conditions in Eq. (18) are performed on a shrinking sheet. Merkin^45^ was the first to propose the stability analysis for the dual solutions and showed that a positive eigenvalue in a dual solution is more stable and dependable than a negative eigenvalue. Next, his work was carried out by Harris et al.^46^, and, recently Hamid et al.^47^, Roşca et al.^48^ who studied the stability of the dual solution on Casson fluid flow on stretching sheet and Khashi’ie et al.^49^ for the axisymmetric flow of a hybrid nanofluid on a radially permeable stretching/shrinking sheet, respectively.

Consider the unsteady Casson fluid flow with the time variable $\varepsilon = r_{1}^{2} bt$. Equations (7-8) can be written as

32 $$\frac{{\partial u_{1} }}{\partial t} + u_{1} \frac{{\partial u_{1} }}{{\partial r_{1} }} + w_{1} \frac{{\partial u_{1} }}{{\partial z_{1} }} = \frac{{\mu_{nf} }}{{\rho_{nf} }}\left( {1 + \frac{1}{\Lambda }} \right)\frac{{\partial^{2} u_{1} }}{{\partial z_{1}^{2} }} - \frac{{\sigma_{nf} B^{2} \left( r \right)}}{{\rho_{nf} }}\sin \left( \tau \right)u_{1} .$$

33 $$\frac{{\partial T_{1} }}{\partial t} + u_{1} \frac{{\partial T_{1} }}{{\partial r_{1} }} + w_{1} \frac{{\partial T_{1} }}{{\partial z_{1} }} = \frac{{k_{nf} }}{{\left( {\rho Cp} \right)_{nf} }}\frac{{\partial^{2} T_{1} }}{{\partial z_{1}^{2} }} - \frac{1}{{\left( {\rho Cp} \right)_{nf} }}\frac{{\partial q_{r} }}{{\partial z_{1} }}.$$

By incorporating Eqs. (7–8) we introduce the new dimensionless variable

34 $$\begin{gathered} u_{1} = br_{1}^{3} F^{\prime}\left( {\xi ,\varepsilon } \right){,}\quad w_{1} = - \sqrt {b\upsilon_{f} } r_{1} \left( {3F\left( {\xi ,\varepsilon } \right) + \xi F^{\prime}\left( {\xi ,\varepsilon } \right)} \right), \hfill \\ \xi = \sqrt {\frac{b}{{\upsilon_{f} }}} z_{1} r_{1} ,\quad \theta \left( {\xi ,\varepsilon } \right) = \frac{{T_{1} - T_{\infty } }}{{T_{w} - T_{\infty } }},\quad \varepsilon = r_{1} bt, \hfill \\ \end{gathered}$$

and by adding the new similarity variable to Eq. (34) and taking $\tau =\frac{\pi }{2}, {\text{then}}\mathit{sin}\left(\tau \right)=1$, the following system of Equations is obtained, as

35 $$A_{1} \left( {1 + \frac{1}{\Lambda }} \right)\frac{{\partial^{3} F}}{{\partial \xi^{3} }} + 3A_{2} F\frac{{\partial^{2} F}}{{\partial \xi^{2} }} - 3A_{2} \left( {\frac{\partial F}{{\partial \xi }}} \right)^{2} - A_{5} M\frac{\partial F}{{\partial \xi }} - A_{2} \frac{{\partial^{2} F}}{\partial \xi \,\partial \varepsilon } = 0,$$

36 $$\left( {A_{4} + N_{r} } \right)\frac{{\partial^{2} \theta }}{{\partial \xi^{2} }}^{\prime\prime} + 3A_{3} \Pr F\frac{\partial \theta }{{\partial \xi }} - A_{3} \Pr \frac{\partial \theta }{{\partial \varepsilon }} = 0,$$

and the boundary condition becomes

37 $$\left\{ \begin{gathered} \frac{\partial F}{{\partial \xi }} = d,\quad F\left( {\xi ,\varepsilon } \right) = V_{c} ,\quad \theta \left( {\xi ,\varepsilon } \right) = 1,\quad {\text{at}}\quad \xi = 0, \hfill \\ \frac{\partial F}{{\partial \xi }} \to 0,\quad \theta \left( {\xi ,\varepsilon } \right) = 0,\quad {\text{as}}\quad \xi \to \infty . \hfill \\ \end{gathered} \right.$$

Equation (36) is introduced by using the notion in Refs.^43,44^ to test the stability of the dual solutions by considering $F\left(\xi \right)={F}_{0}\left(\xi \right)$ and , and in the current analysis we have obtained the unsteady form of the Casson fluid on the shrinking sheet as

38 $$\left\{ {\begin{array}{*{20}c} {F\left( {\xi ,\varepsilon } \right) = F_{0} \left( \xi \right) + Exp\left[ { - \gamma \xi } \right]f\left( {\xi ,\varepsilon } \right),} \\ {\theta \left( {\xi ,\varepsilon } \right) = \theta_{0} \left( \xi \right) + Exp\left[ { - \gamma \xi } \right]\varphi \left( {\xi ,\varepsilon } \right),} \\ \end{array} } \right.$$

where $\gamma$ is known as unidentified eigenvalue and $f\left(\xi ,\varepsilon \right) \, {\text{and}} \, \varphi \left(\xi ,\varepsilon \right)$ are assumed to be smaller than ${F}_{0}\left(\xi \right)\text{ and}{ \theta }_{0}\left(\xi \right)$. The unstable solution of Eqs. (33–35) results in an infinite value of $\left(\gamma \right)$ eigenvalue. Initial instability increase relates to a negative eigenvalue, but initial instability decline relates to a positive eigenvalue which implies that the Casson fluid flow over the surface is stable. The unsteady form of the system of differential equations along with boundary condition follow,

39 $$A_{1} \left( {1 + \frac{1}{\Lambda }} \right)\frac{{\partial^{3} f}}{{\partial \xi^{3} }} + 3A_{2} F_{0} \frac{{\partial^{2} f}}{{\partial \xi^{2} }} + 3A_{2} f\,F_{0} ^{\prime\prime} - A_{2} \left( {6F_{0} ^{\prime} - \gamma } \right)\frac{\partial f}{{\partial \xi }} - A_{5} M\frac{\partial f}{{\partial \xi }} - A_{2} \frac{{\partial^{2} F}}{\partial \xi \,\partial \varepsilon } = 0,$$

40 $$\left( {A_{4} + N_{r} } \right)\frac{{\partial^{2} \varphi }}{{\partial \xi^{2} }} + 3A_{3} \Pr F_{0} \frac{\partial \varphi }{{\partial \xi }} + 3A_{3} \Pr f\,\theta_{0} ^{\prime} + \gamma \varphi - A_{3} \Pr \frac{\partial \varphi }{{\partial \varepsilon }} = 0,$$

41 $$\left\{ \begin{gathered} \frac{\partial f}{{\partial \xi }} = 0,\quad \quad f\left( {\xi ,\varepsilon } \right) = 0,\quad \varphi \left( {\xi ,\varepsilon } \right) = 0,\quad {\text{at}}\quad \xi = 0, \hfill \\ \frac{\partial f}{{\partial \xi }} \to 0,\quad \varphi \left( {\xi ,\varepsilon } \right) \to 0,\quad {\text{as}}\quad \xi \to \infty . \hfill \\ \end{gathered} \right.$$

The generalized linear eigenvalue problem with the boundary conditions is derived by substituting $\varepsilon = 0$ in Eqs. (37–39), which represents the solution in a stable state.

42 $$A_{1} \left( {1 + \frac{1}{\Lambda }} \right)f_{0}^{\prime \prime \prime } + 3A_{2} F_{0} f_{0}^{\prime \prime } + 3A_{2} f_{0} \,F_{0}^{\prime \prime } - A_{2} \left( {6F_{0}^{\prime } - \gamma } \right)f_{0}^{\prime } - A_{5} M\,f_{0}^{\prime } = 0,$$

43 $$\left( {A_{4} + N_{r} } \right)\varphi_{0}^{\prime \prime } + 3A_{3} \Pr F_{0} \varphi_{0}^{\prime } + 3A_{3} \Pr f_{0} \,\theta_{0}^{\prime } + \gamma \varphi_{0} = 0,$$

44 $$\left\{ \begin{gathered} \frac{{\partial f_{0} }}{\partial \xi } = 0,\quad f_{0} \left( \xi \right) = 0,\quad \varphi_{0} \left( \xi \right) = 0,\quad {\text{at}}\quad \xi = 0, \hfill \\ \frac{{\partial f_{0} }}{\partial \xi } \to 0,\quad \varphi_{0} \left( \xi \right) \to 0,\quad \quad \quad {\text{as}}\quad \xi \to \infty . \hfill \\ \end{gathered} \right.$$

The stability of the relevant solution ${F}_{0}\text{ and }{\theta }_{0}$ is determined by calculating the least value of the eigenvalue for the specific value of $\Lambda ,\,\,N_{r} ,\,\,M,\,\,\gamma ,\,\,{\text{P}}_{r}$. In this investigation, we swapped out the equivalent boundary condition at free stream $f_{0} ^{\prime} \to 0$ as $\xi \to \infty$ for $f_{0} ^{\prime\prime}\left( 0 \right) = 1.$

### Ethical approval

This article does not contain any studies with human participants or animals performed by any of the authors.

## Results and discussion

In the present paper, we have argued on the closed form of the exact analytical dual branch solution for SWCNTs and MWCNTs. Graphical results for various values of ${V}_{c}$, M, Nr, $\phi$ and d, are to be shown next. The computations are made for $\phi =0.1, {V}_{c}=2.4, d=-1.1, M=0.1, {N}_{r}=1$ and $\Lambda =1,$ while the thermophysical properties of water and CNTs are taken from Table 1. Note that the Prandtl number of the base fluid (water) is equal to 6.2.

The solution obtained in this analysis is in the form of upper and lower branch solution which are represented graphically in first and second solution, respectively. From the results obtained we come in conclusion that the upper branch solution is more stable than the lower branch solution.

### Velocity and temperature profiles

**Figure 3:**
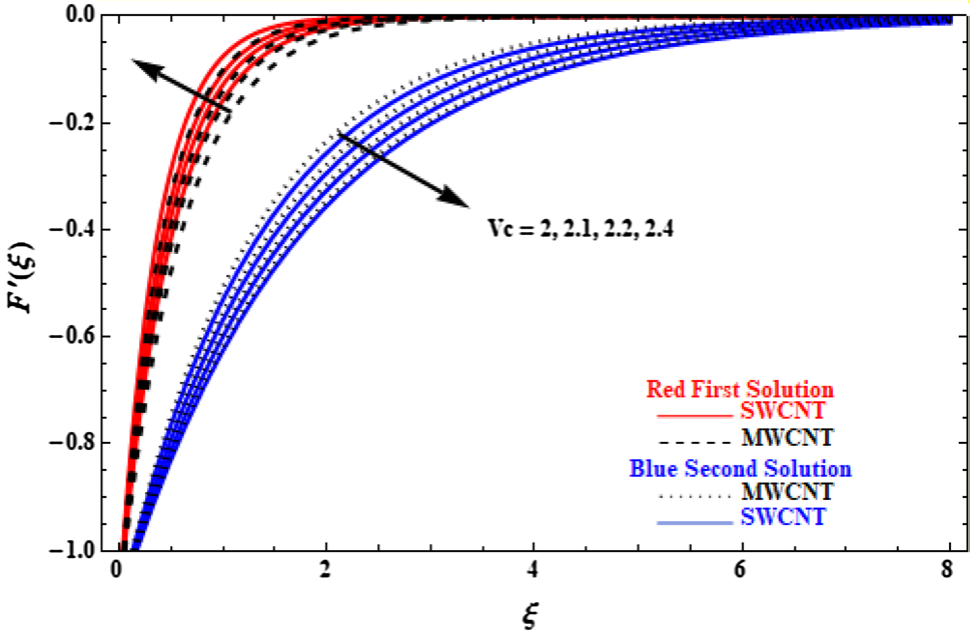
Plot of velocity $F^{\prime}\left( \xi \right)$ versus $\xi$ for various $V_{c}$ values.

**Figure 4:**
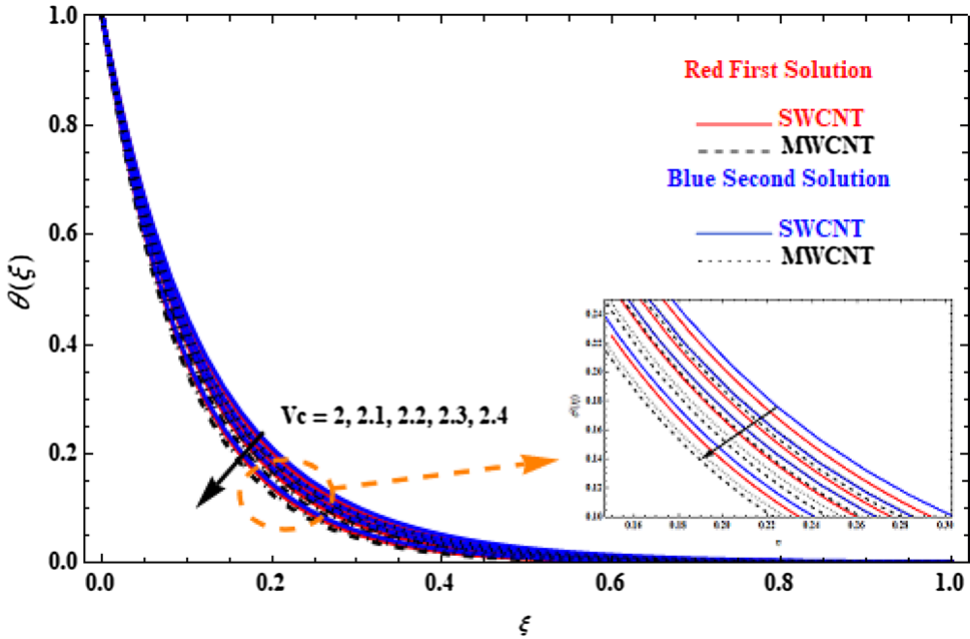
Plot of temperature $\theta \left( \xi \right)$ versus $\xi$ for various $V_{c}$ values.

Figures 3 and 4 show the effect of ${V}_{c}$ on the velocity and temperature profiles of SWCNT and MWCNT, respectively. It is observed that the velocity of the upper branch solution, both for SWCNT and MWCNT, increases with as ${V}_{c}$ increases, too. Moreover, SWCNT attains higher velocity values compared to MWCNT. In contradistinction, the reverse behaviour is observed for the lower branch solution, where the velocity decreases with as ${V}_{c}$ increases. This is due to the fact that by increasing ${V}_{c}$, the fluid near the surface is being pulled up more quickly, producing the faster fluid velocity. Another point worth mentioning is that the temperatures of SWCNTs and MWCNTs, for both upper and lower branch solutions, present similar behaviour, as they decrease with increasing ${V}_{c}$. Also, MWCNTs have overall achieved lower temperature compared to SWCNTs, owing to fluid flow over the permeable plate, which removes the highly heated fluid.

**Figure 5:**
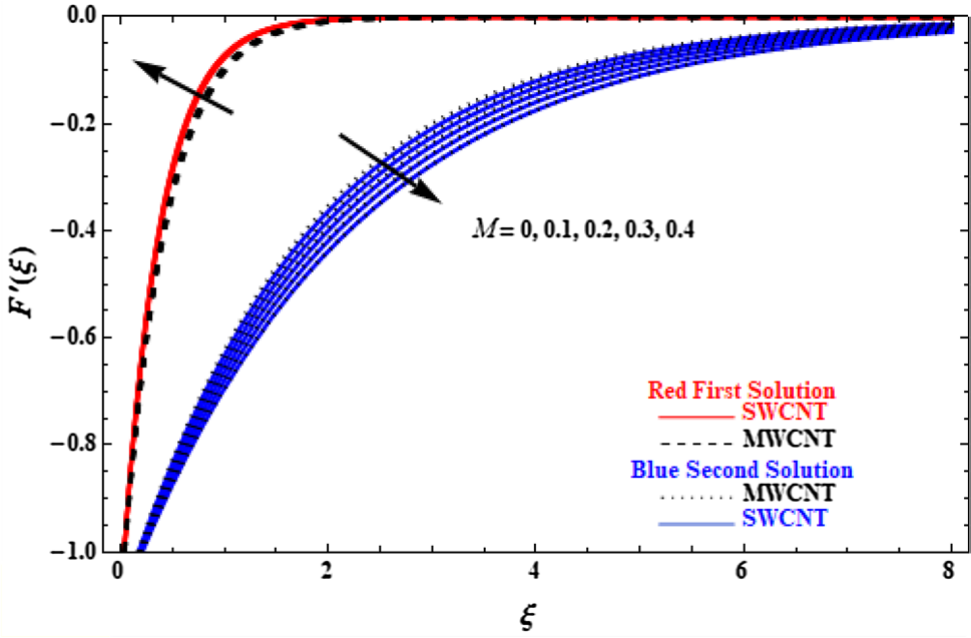
Plot of velocity $F^{\prime}\left( \xi \right)$ versus $\xi$ for various values of $M$.

**Figure 6:**
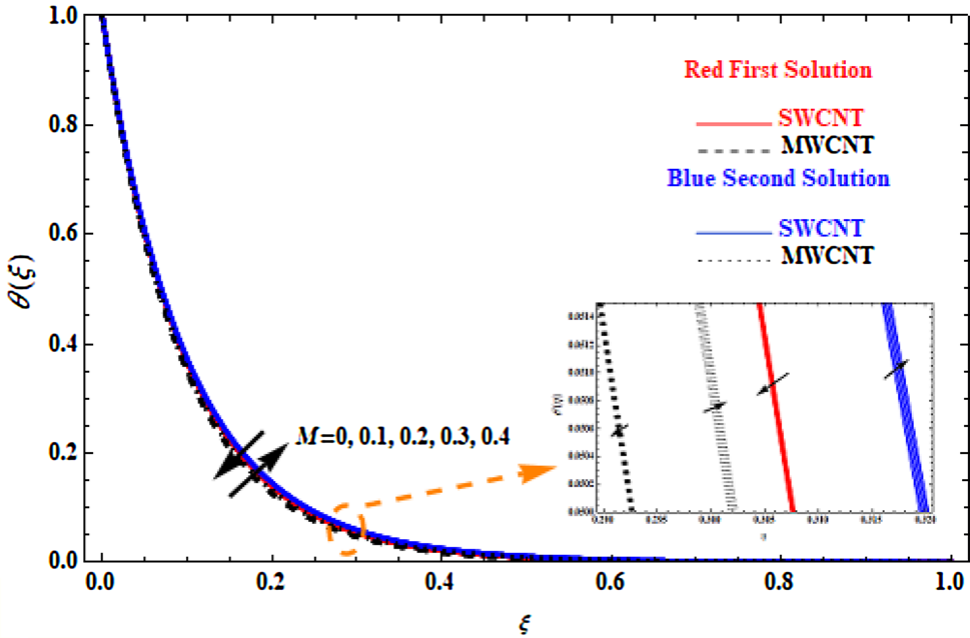
Plot of temperature $\theta \left(\xi \right)$ versus $\xi$ for various values of $M$.

The effect of the application of the inclined magnetic field $M$ on velocity and temperature profiles is depicted in Figs. 5 and 6. Different behaviour is observed between SWCNT and MWCNT. In Fig. 5 (upper branch solution), for higher values of $M$, the velocity of both SWCNT and MWCNT increases, with SWCNT reaching slightly higher values compared to MWCNT. However, in the lower branch, the velocity of SWCNT and MWCNT decreases as *M* increases, while SWCNT has smaller velocity values compared to MWCNT. In Fig. 6, the temperature of the upper branch solutions is decreased when $M$ increases, but opposite behaviour is observed in the second branch solution, since MWCNT has generally lower temperatures compared to SWCNT. This is attributed to the fact that nanoparticles are greatly influenced by the magnetic field, and higher values of *M* lead to velocity increase and the fluid temperature decrease.

**Figure 7:**
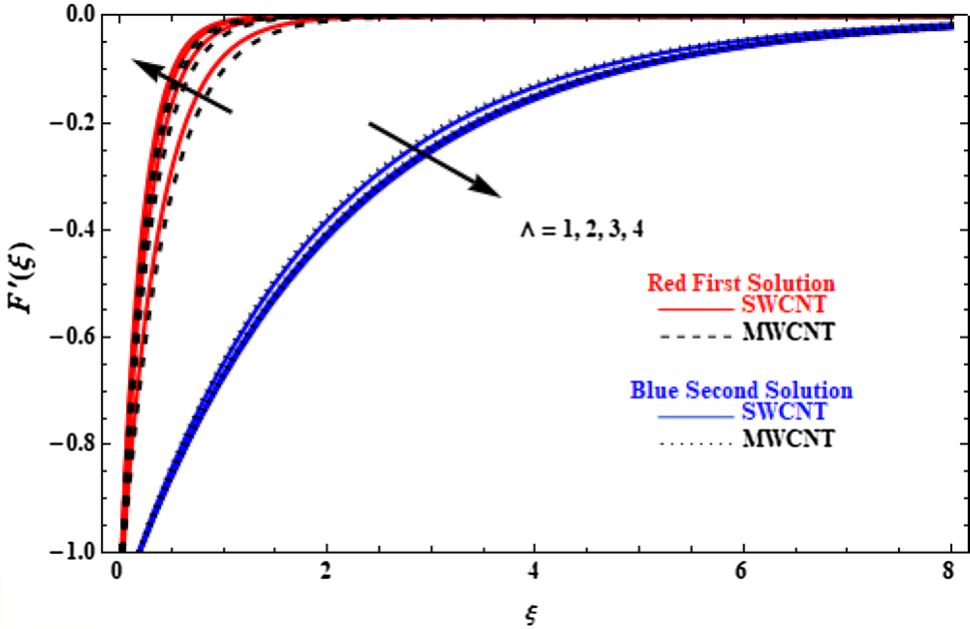
Plot of velocity $F^{\prime}\left( \xi \right)$ versus $\xi$ for various values of  $\Lambda$.

**Figure 8:**
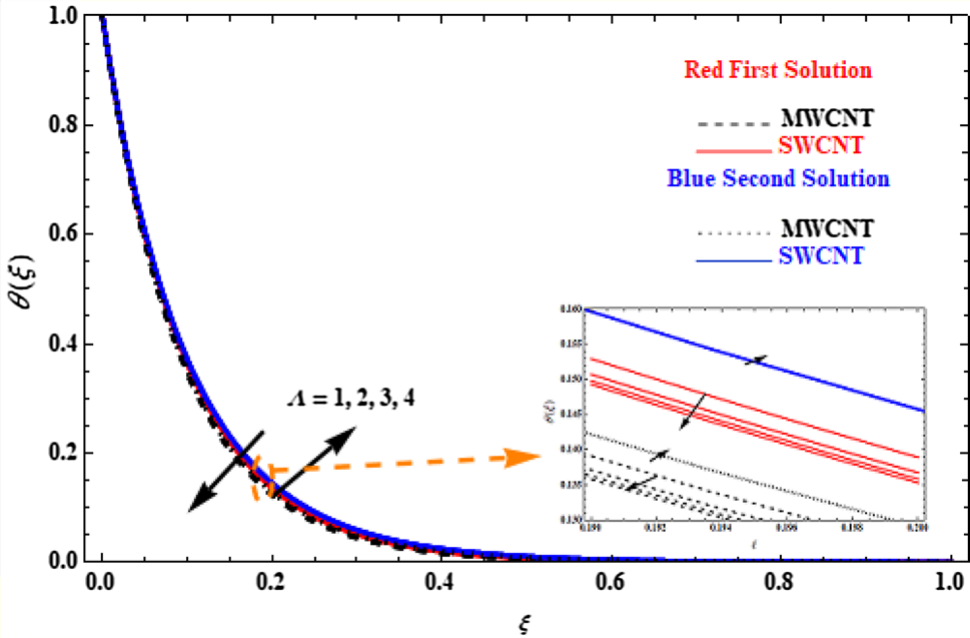
Plot of temperature $\theta \left(\xi \right)$ versus $\xi$ for various values of  $\Lambda$.

Velocity and temperature profiles of SWCT and MWCNT are further examined on the effect of the Casson parameter $\Lambda$ in Figs. 7 and 8. It is shown that the upper branch solution velocity of SWCNT and MWCNT increases when $\Lambda$ increases, with MWCNT cases presenting lower velocity values than SWCNT, and, the reverse effect is observed in the lower branch solution (Fig. 7). This is because the higher values of $\Lambda$ affects the dynamic viscosity, which results in a decrease in the yield stress and, thus, causes a resistance force that opposes to fluid mobility. The effect of $\Lambda$ on temperature is small (Fig. 8). All upper branch solutions present smaller temperature with increasing $\Lambda$, while lower branch solutions present the reverse behaviour when $\Lambda$ increases.

**Figure 9:**
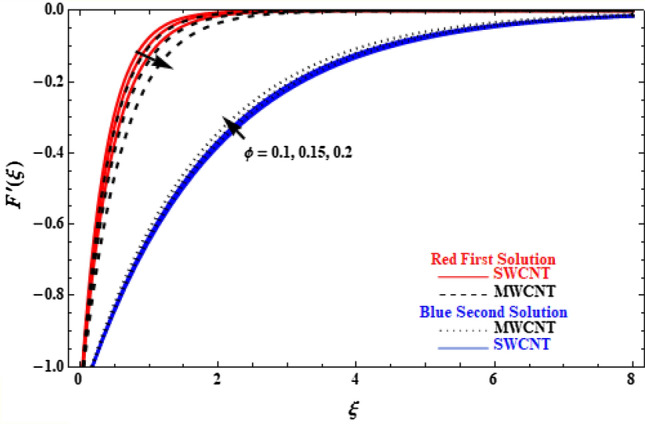
Plot of velocity $F^{\prime}\left( \xi \right)$ versus $\xi$ for various values of $\phi$.

**Figure 10:**
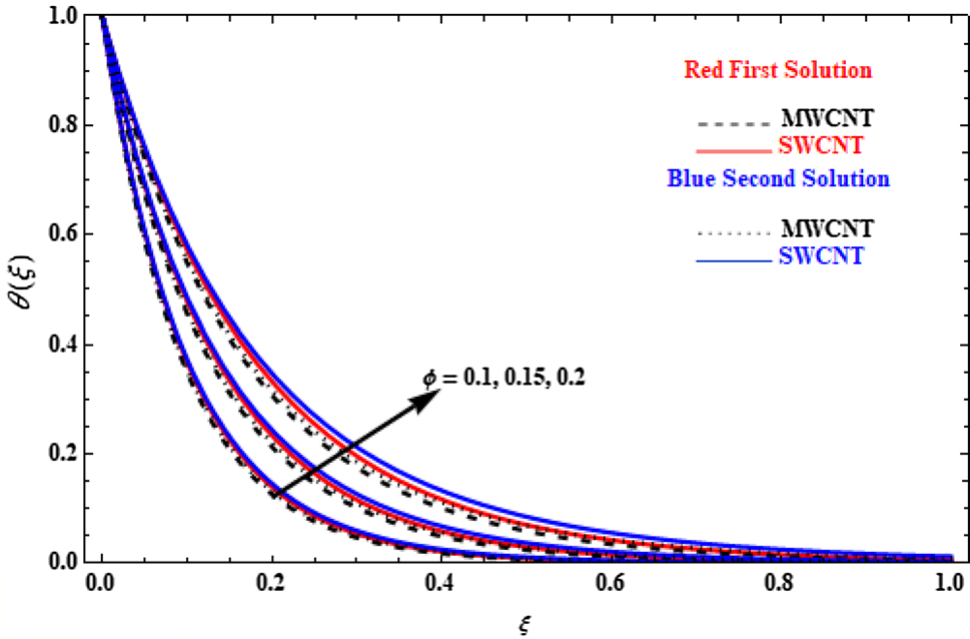
Plot of temperature $\theta \left(\xi \right)$ versus $\xi$ for various values of  $\phi$.

The investigation of the variation of nanoparticle volume fraction, $\phi$, is presented in Figs. 9 and 10. In Fig. 9, as $\phi$ varies from 0.1 to 0.15 and 0.2, the velocity of the upper branch solution decreases, while velocity for the lower branch increases. We also observe higher SWCNT velocities on the upper branch and smaller on the lower branch, compared to MWCNT. It is of importance to note that $\phi$ has significant effect on temperature values, since temperature of SWCNT and MWCNT of both upper and lower branch solutions increase with increasing $\phi$. Moreover, SWCNT has given higher temperature values compared to MWCNT (Fig. 10). This is due to the addition of more nanoparticles which serve as heat transfer agents and causes temperature to rise with increasing value of $\phi$.

**Figure 11:**
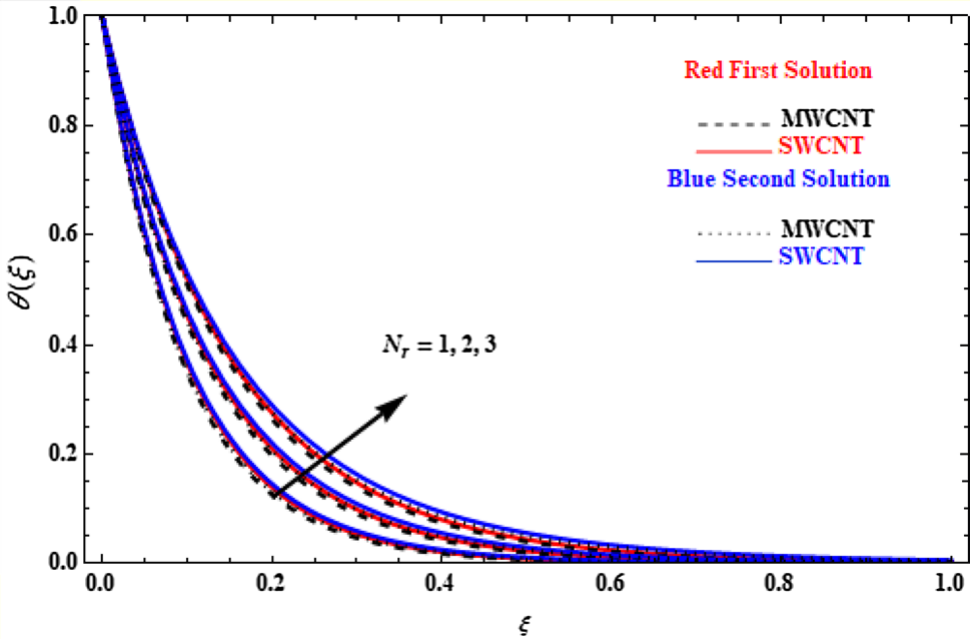
Plot of temperature $\theta \left(\xi \right)$ versus $\xi$ for various values of  ${N}_{r}$.

The effect of the thermal radiation, ${N}_{r}$, on the temperature profiles for our model is depicted in Fig. 11. All profiles present higher values with increasing ${N}_{r}$, while, the SWCNT cases have slightly higher values than MWCNT. This is because the thermal radiation transfers more heat than any other parameter shown before. In physical terms, the thermal conductivity of CNTs increases with the increase of the radiation parameter, and, as a result, the thermal boundary-layer thickness and the profiles of temperature increase.

**Figure 12:**
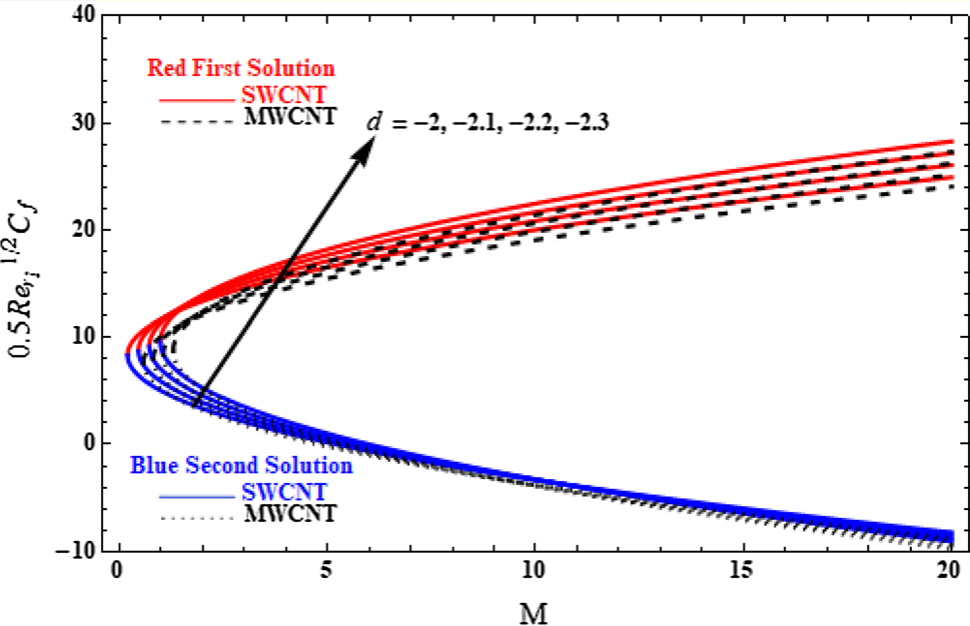
Plot of $0.5{\mathit{Re}}_{{r}_{1}}^{1/2}{C}_{f}$ versus $M$ for various values of $d$.

In the presence of SWCNTs and MWCNTs, the skin friction coefficient versus *M* (Fig. 12) has shown that multiple solution exists only for the shrinking of the sheet. With increasing shrinking parameter values, the skin friction coefficient increases in both branches of the solution. In most cases, this occurs because the motion of the fluid is decreased when the shrinking parameter is increased. As a result, the velocity and skin friction obey to the inverse proportional relationship. Hence, this fact leads to the outcome that skin friction coefficient increases in presence of CNTs.

**Figure 13:**
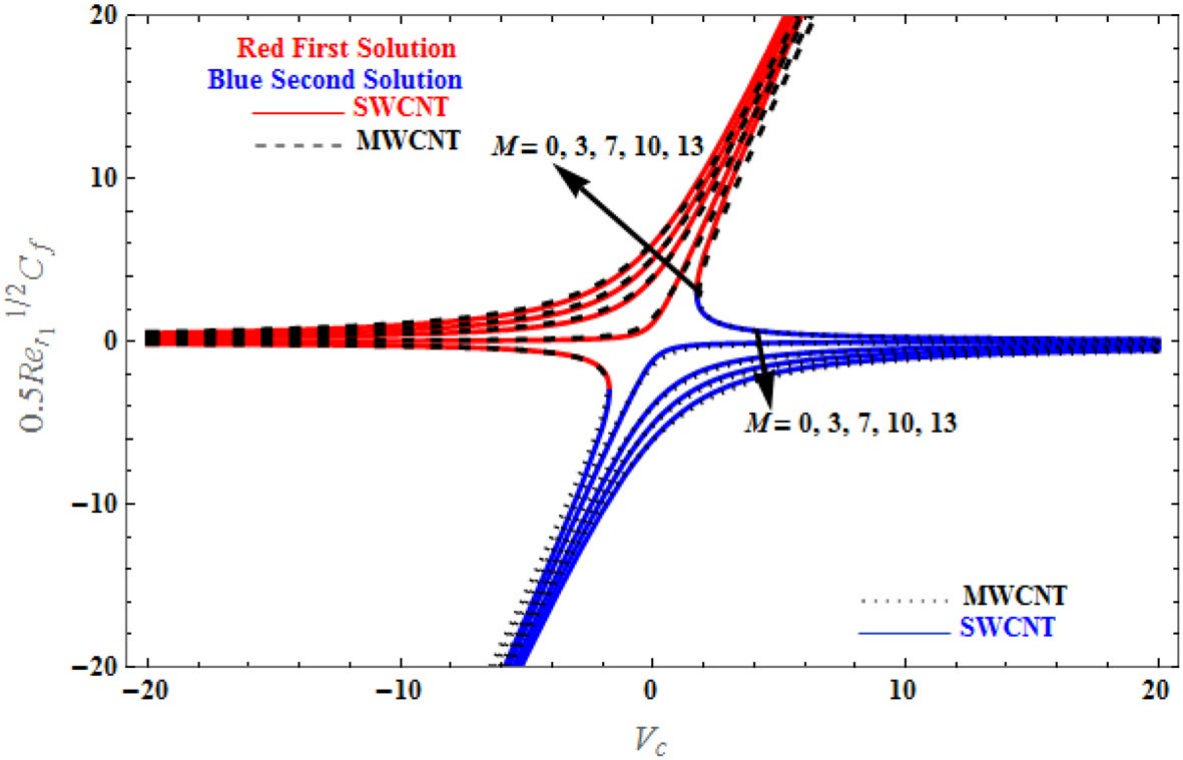
Plot of $0.5{\mathit{Re}}_{{r}_{1}}^{1/2}{C}_{f}$ versus ${V}_{c}$ for various values of $M$.

Figure 13 presents the dual solution for the skin friction coefficient versus ${V}_{c}$, for various values of *M*. The skin friction coefficient of SWCNTs and MWCNTs decreases as the suction parameter decreases and increases when the injection parameter increases, as a consequence of increasing the magnetic field *M*. It also has to be specified that the skin friction coefficient approaches zero value in case of high injection speed, while it increases exponentially at higher suction velocity. Furthermore, greater *M* values have an incremental effect on the shear stress. The behaviour of skin friction coefficient on SWCNTs and MWCNTs for various ${V}_{c}$ values can be summarized by the fact that suction causes greater velocity gradients at the surface, whereas injection minimises them.

**Figure 14:**
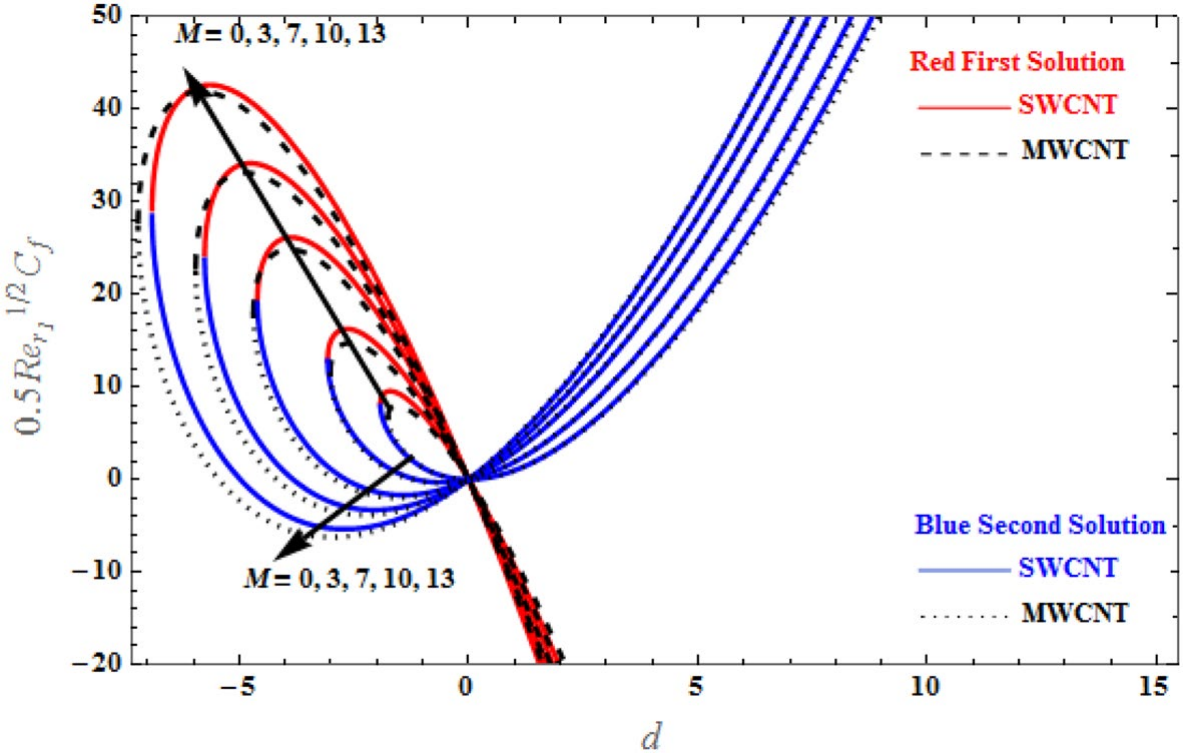
Plot of $0.5{Re}_{{r}_{1}}^{1/2}{C}_{f}$ versus $d$ for various values of $M$.

Figure 14 presents the dual solution for skin friction coefficient versus *d* for various *M* values. The skin friction coefficient of SWCNTs and MWCNTs increases as $d$ decreases because the velocity gradients are larger when the plate decreases quicker or is stretched at a slower pace. Hence, at large values of the magnetic field, the skin friction coefficients of SWCNTs and MWCNTs increases, and this is due to the greater fluid velocity observed when the magnetic field value increases. Thus, the skin friction coefficients of SWCNTs and MWCNTs are proportionally increasing with the magnetic field.

### Validation

The research has revealed that the SWCNTs achieve higher temperature then MWCNTs on non-Newtonian Casson fluid model on porous shrinking sheet. The dual solution obtained opens the pathway to future studies of Casson fluid flows under the impact of an inclined MHD, radiation and mass transpiration. The velocity behaviour in the absence of Casson fluid and $M=0$ in the presence of stretching plate results are converted^14^, while $\mathit{sin}\left(\tau \right)=1$ in the presence of hybrid nano particles leads to the results of Khan et al.^34^.

Table 2 presents relevant works from the literature for comparison.

**Table 2:** Comparison to existing methods.

Authors	Fluid	Value of $\beta$
Crane^14^	Newtonian	$\beta = 1$
Khan et al.^34^	Newtonian	$f\left( \eta \right) = f_{w} + \frac{\lambda }{{\beta_{\alpha } }}\left( {1 - e^{{ - \beta_{\alpha } \xi }} } \right),$ $\frac{{\mu_{Hnf} }}{{\mu_{f} }}\beta_{\alpha }^{2} - 3\frac{{\rho_{Hnf} }}{{\rho_{f} }}\beta_{\alpha } f_{w} - \left( {3\frac{{\rho_{Hnf} }}{{\rho_{f} }}\lambda + \frac{{\sigma_{Hnf} }}{{\sigma_{f} }}M} \right) = 0,$ and the roots are $\beta_{\alpha } = \frac{{3\frac{{\rho_{Hnf} }}{{\rho_{f} }}f_{w} \pm \sqrt {9f_{w}^{2} \left( {\frac{{\rho_{Hnf} }}{{\rho_{f} }}} \right)^{2} + 4\frac{{\mu_{Hnf} }}{{\mu_{f} }}\left( {3\frac{{\rho_{Hnf} }}{{\rho_{f} }}\lambda + \frac{{\sigma_{Hnf} }}{{\sigma_{f} }}M} \right)} }}{{2\frac{{\mu_{Hnf} }}{{\mu_{f} }}}}$
		Skin friction $0.5{\text{Re}}_{{r_{1} }}^{1/2} C_{f} = \frac{{\mu_{Hnf} }}{{\mu_{f} }}F^{\prime\prime}\left( 0 \right)$
Present work	Non-Newtonian (Casson fluid)	$F\left( \xi \right) = V_{c} + \frac{d}{\beta }\left( {1 - Exp\left[ { - \beta \xi } \right]} \right).$ $A_{1} \left( {1 + \frac{1}{\Lambda }} \right)\beta^{2} - 3A_{2} V_{c} \beta - \left( {A_{5} M + 3A_{2} d} \right) = 0,$ and the roots are $\beta = \frac{{3V_{c} A_{2} \pm \sqrt {9V_{c}^{2} A_{2}^{2} - 4\left( {1 + \frac{1}{\Lambda }} \right)A_{1} \left( { - 3dA_{2} - MA_{5} } \right)} }}{{2\left( {1 + \frac{1}{\Lambda }} \right)A_{1} }}$
		Skin friction $0.5{\text{Re}}_{{r_{1} }}^{1/2} C_{f} = \frac{{\mu_{nf} \left( {1 + \frac{1}{\Lambda }} \right)}}{{\mu_{f} }}F^{\prime\prime}\left( 0 \right)$

Table 3 presents the comparison of the stability analysis performed here with other works that have conducted the stability analysis with a time variable. Khashi'ie et al.^49^ and Hamid et al.^47^ have taken $\tau =ct$, while, in the present work, we have considered $\varepsilon ={{r}_{1}}^{2}bt$.

**Table 3:** Comparison of the stability analysis with similar works.

Approaches	Fluids	Stability analysis (Stable /unstable)
Hamid et al.^47^	Non-Newtonian	$\left( {1 + \frac{1}{\Lambda }} \right)\frac{{\partial^{3} F}}{{\partial \eta^{3} }} + F_{0} \frac{{\partial^{2} F}}{{\partial \eta^{2} }} + \left( {\frac{\partial F}{{\partial \eta }}} \right)^{2} - M\frac{\partial F}{{\partial \eta }} - \frac{{\partial^{2} F}}{\partial \tau \partial \mu \eta } = 0,$ $\frac{1}{{Pr\left( {1 + R_{d} } \right)\frac{{\partial^{2} \varphi }}{{\partial \eta^{2} }}_{0} \frac{\partial \varphi }{{\partial \eta }}\frac{\partial \varphi }{{\partial \varepsilon }}}}$
Khashi'ie et al.^49^	Newtonian	$\frac{{\frac{{\mu_{hnf} }}{{\mu_{f} }}}}{{\frac{{\rho_{hnf} }}{{\rho_{f} }}}}\frac{{\partial^{3} F}}{{\partial \eta^{3} }} + 2f_{0} \frac{{\partial^{2} F}}{{\partial \eta^{2} }} + 2F\frac{{\partial^{2} f_{0} }}{{\partial \eta^{2} }} - \left( {2f_{0}^{^{\prime}} - \gamma + \left( {\frac{{\frac{{\sigma_{hnf} }}{{\sigma_{f} }}}}{{\frac{{\rho_{hnf} }}{{\rho_{f} }}}}} \right)M} \right)\frac{\partial F}{{\partial \eta }} = 0,$ $\frac{1}{{Pr\frac{{\left( \kappa \right)_{hnf} /\left( \kappa \right)_{f} }}{{\left( {\rho Cp} \right)_{hnf} /\left( {\rho Cp} \right)_{f} }}\frac{{\partial^{2} G}}{{\partial \eta^{2} }}\frac{{\partial \theta_{0} }}{\partial \eta }_{0} \frac{\partial G}{{\partial \eta }}_{0} \frac{\partial F}{{\partial \eta }}}}$ $- \left( {2\frac{{\partial f_{0} }}{\partial \eta } - \gamma } \right)G + 2\left( {\frac{{\sigma_{hnf} /\sigma_{f} }}{{\left( {\rho Cp} \right)_{hnf} /\left( {\rho Cp} \right)_{f} }}} \right)EcM\frac{{\partial f_{0} }}{\partial \eta }\frac{\partial F}{{\partial \eta }} = 0,$
Present work	Non-Newtonian	$$A_{1} \left( {1 + \frac{1}{\Lambda }} \right)\frac{{\partial^{3} f}}{{\partial \xi^{3} }} + 3A_{2} F_{0} \frac{{\partial^{2} f}}{{\partial \xi^{2} }} + 3A_{2} fF_{0} ^{\prime\prime} - A_{2} \left( {6F_{0} ^{\prime} - \gamma } \right)\frac{\partial f}{{\partial \xi }}$$$$- A_{5} M\frac{\partial f}{{\partial \xi }} - A_{2} \frac{{\partial^{2} F}}{\partial \xi \partial \varepsilon } = 0,$$ $$\left( {A_{4} + N_{r} } \right)\frac{{\partial^{2} \varphi }}{{\partial \xi^{2} }} + 3A_{3} \Pr F_{0} \frac{\partial \varphi }{{\partial \xi }} + 3A_{3} \Pr f\theta_{0} ^{\prime} + \gamma \varphi - A_{3} \Pr \frac{\partial \varphi }{{\partial \varepsilon }} = 0,$$

Finally, in Table 4, values of skin friction coefficients both for the upper and lower branch solutions, related to Fig. 12, are presented.

**Table 4:** Skin friction coefficient,${\text{Re}}_{{r_{1} }}^{1/2} C_{f}$, for various values of *d* and *M*.

$M$	First solution (upper branch solution)				Second solution (lower branch solution)			
	$d=-2$	$d=-2.1$	$d=-2.2$	$d=-2.3$	$d=-2$	$d=-2.1$	$d=-2.2$	$d=-2.3$
SWCNT								
0	8.35744	8.77532	9.19319	9.61106	8.35744	8.77532	9.19319	9.61106
5	16.5273	17.1174	17.6778	18.2069	0.187634	0.433234	0.708546	1.01525
10	20.0258	20.8629	21.682	22.4826	− 3.31094	− 3.31227	− 3.29559	− 3.26047
15	22.6947	23.6961	24.6835	25.6568	− 5.97979	− 6.14544	− 6.29713	− 6.43465
20	24.9394	26.0712	27.1913	28.2994	− 8.22452	− 8.52059	− 8.80487	− 9.07726
MWCNT								
0	7.63535	8.01712	8.39889	8.78065	7.63535	8.01712	8.39889	8.78065
5	15.4751	16.0240	16.5447	17.0355	− 0.20443	0.010218	0.253092	0.525776
10	19.0751	19.8759	20.6600	21.4271	− 3.80438	− 3.84162	− 3.86220	− 3.86575
15	21.7871	22.7531	23.7062	24.6463	− 6.51642	− 6.71886	− 6.90846	− 7.08503
20	24.0572	25.1539	26.2397	27.3146	− 8.78652	− 9.11964	− 9.44194	− 9.75333

## Conclusion

This paper has presented a detailed investigation of the boundary-layer flow and heat transfer properties implied by water-based CNTs over a permeable shrinking sheet, affected by the parameters of the Casson fluid, an inclined magnetic field and thermal radiation effects. The exact solutions for velocity and temperature have been obtained with aid of the Gamma function. The analytical results have been graphically illustrated versus various dimensionless physical characteristics that have been found to affect velocity, shear stress, and temperature distribution.

- The velocity profile of the upper branch solution of SWCNT and MWCNT nanoparticles has obtained higher values when mass transpiration $\left({V}_{c}\right)$ and magnetic field $\left(M\right)$ increase, but the opposite behaviour is observed for the lower branch solutions. Temperature values, on the other hand, have shown similar decreasing behaviour for both branches.
- The velocity profile also increases when $\Lambda$ increases. The reverse behaviour appears for the lower branch solutions, while temperature is clearly decreased for upper branch solutions and increased for lower branch solutions.
- The fluid velocity of upper and lower branch solutions of all CNT nanoparticles decreases and increases, respectively. Furthermore, the temperature of both branch solutions obtains higher values when $\phi$ increases.
- The temperature for both branch solutions of CNT nanoparticles increases when the thermal radiation *N*_*r*_ increases. Moreover, it is observed that SWCNTs achieve higher temperatures compared to MWCNTs.
- The skin friction coefficient both carbon nanotubes increases for decreasing d and increases with M.

To conclude, we believe that the main findings of this analytical investigation can find field of applicability in various engineering and industrial applications, concerning medical applications, micro-fluidic devices, nanofluidic pumps, and space technology, among others.

## Data Availability

The datasets used and/or analysed during the current study available from the corresponding author on reasonable request.

## Latin symbols

$${A}_{1},{A}_{2},{A}_{3},{A}_{4},{A}_{5}$$: Constants
${B}_{0}$: Magnetic field strength $\left[\mathrm{Tesla}\right]$
${C}_{P}$: Specific heat at constant pressure
${H}_{0}$: Applied magnetic field $\left[{\mathrm{wm}}^{-2}\right]$
${k}^{*}$: Mean absorption coefficient
*M*: Magnetic field
$${N}_{r}$$: Radiation parameter
Pr: Prandtl number
${q}_{r}$: Radiative heat flux
${q}_{w}$: Local heat flux at the wall
$${T}_{1}$$: Temperature
$${V}_{c}$$: Mass transformation
$\left({r}_{1,}\xi \right)$: Cylindrical coordinate axes
$\left({r}_{1},\xi ,{z}_{1}\right);$: Cartesian cylindrical coordinate axes

## Greek symbols

$$\Lambda$$: Casson parameter
*κ*: Thermal conductivity of fluid $\left[{\mathrm{WK{g}}}^{-1}{\mathrm{K}}^{-1}\right]$
*ξ*: Similarity variable
$${\mu }_{f}$$: Dynamic viscosity $$\left[{\mathrm{Ns{m}}}^{-1}\right]$$
$$\nu$$: Kinematic viscosity $$\left[{\mathrm{m}}^{2}{\mathrm{s}}^{-1}\right]$$
*ρ*: Density $$\left[{\mathrm{Kg{m}}}^{-3}\right]$$
*σ*: Electrical conductivity $$\left[{\mathrm{s/m}}\right]$$
${\sigma }^{*}$: Stefan–Boltzmann constant $\left[{\mathrm{W{m}}}^{-2}{\mathrm{K}}^{-2}\right]$
$$\Gamma$$: Gamma function
*φ*: Nanoparticle volume fraction
*ψ*: Stream function
*τ*: Angle of inclination of magnetic field

## Subscripts

$$f$$: Base fluid
$$nf$$: Nanofluid

## Abbreviations

MHD: Magnetohydrodynamics
CNT: Carbon nanotube
SWCNT: Single wall carbon nanotube
MWCNT: Multi-walled carbon nanotube
LT: Laplace transformation
